# Efficacy and safety of Xiaofeng powder in the treatment of chronic urticaria: A systematic review and meta-analysis

**DOI:** 10.1097/MD.0000000000037305

**Published:** 2024-03-22

**Authors:** Chenhao Bi, Yuqi Jia, Fengqin Wei

**Affiliations:** a College of Traditional Chinese Medicine, Shandong University of Traditional Chinese Medicine, Jinan, China; b College of Acupuncture and Massage, Shandong University of Traditional Chinese Medicine, Jinan, China.

**Keywords:** chronic urticaria, meta-analysis, systematic review, Xiaofeng powder

## Abstract

**Background:**

Chronic urticaria is a group of skin diseases characterized by pruritus and/or vascular oedema and belongs to the category of “addictive rash” in Traditional Chinese Medicine, and its aetiology is closely related to wind evil. Antihistamines are often used in treatment. Although they have certain effects, they also easily cause disease recurrence. Xiaofeng powder treats this disease has a significant effect in improving the disease state and reducing the recurrence rate. However, there is a lack of evidencebased research. This study to systematically evaluate the clinical efficacy of modified Xiaofeng powder in the treatment of chronic urticaria (CU).

**Methods:**

Computer searches of Chinese databases such as China National Knowledge Infrastructure, China Scientific Journal Database, China Biomedical Literature Database, and WanFang Date and foreign databases such as PubMed and the Web of Science were performed. We retrieved published clinical randomized controlled trials of Xiaofeng powder in the treatment of CU from the establishment of the databases to November 2023. The data were extracted from clinical trials that met the inclusion criteria of this study, and the quality was evaluated through the Cochrane Handbook of Systematic Reviews 5.1.0. Finally, a meta-analysis was performed using RevMan 5.3 statistical software.

**Results:**

A total of 11 randomized controlled trials involving 1076 patients were included. The cure rate odds ratio (OR) and 95% confidence interval (CI; shown in brackets) were 2.11 [1.45, 3.07]; the total effective rate OR and CI were 2.42 [1.60, 3.68]; the recurrence rate OR and CI were 0.22 [0.15, 0.34]; the adverse reaction rate OR and CI were 0.23 [0.12, 0.45]; and the mean weighted mean difference (MD) and 95% CI (shown in brackets) of itching degree, wind mass size, wind mass number and wind mass duration in symptom and sign integrals were −0.70 [−0.73, 0.67], −0.64 [−0.96, 0.31], , −0.72 [−1.23, 0.22], and −0.68 [−1.13, 0.23], , respectively.

**Conclusion:**

The clinical efficacy of modified Xiaofeng powder in the treatment of CU is better than that of antihistamine drugs, with lower adverse reaction and recurrence rates and higher safety. However, the quality of clinical research included is relatively low, and findings need to be confirmed by high-quality research.

## 1. Introduction

Urticaria is a group of skin diseases characterized by pruritus and/or vascular edema.^[1]^ According to the inducing factors and disease course, there are many types of urticaria in the clinic. If the attack time of the wind mass exceeds 6 weeks, it is chronic urticaria (CU).^[2]^ It is reported that urticaria affects approximately 9% to 20% of the population worldwide; the incidence in Asia is higher than that in other regions, and in China, it is approximately 23%.^[3]^ Compared with other skin diseases, urticaria has a longer disease course, and due to its characteristic easy recurrence, the treatment process is prolonged, and difficulties with medication are increased. Antihistamines are often used in treatment. Although they have certain effects, they also easily cause disease recurrence.

CU belongs to the category of “addictive rash” in Traditional Chinese Medicine (TCM), and its etiology is closely related to wind evil. Huangdi Neijing reported, “Wind evil is the first disease,” which means that wind pathogens mostly carry cold, dampness, heat, and other pathogens to the human body. If the body is deficient in healthy qi and defensive qi is not solid, wind pathogens can invade the body according to the situation, resulting in the nutrient deficiencies and defensive qi. As a commonly used prescription in the clinical treatment of this disease in TCM, Xiaofeng powder has a significant effect in improving the disease state and reducing the recurrence rate. However, there is still a lack of systematic evaluation of the efficacy and safety of Xiaofeng powder in the treatment of CU from the perspective of evidence-based medicine. In this paper, a meta-analysis was conducted to provide reference evidence for the clinical treatment of CU.

## 2. Methods

The protocol and registration information are available at https://www.crd.york.ac.uk/prospero/display_record.php?RecordID=223954 (registration number: CRD42021223954). We performed this meta-analysis according to the Preferred Reporting Items for Systematic Reviews and Meta-Analyses statement.

### 2.1. Search strategy

Computer searches included Chinese databases such as China National Knowledge Infrastructure, China Scientific Journal Database, China Biomedical Literature Database, and WanFang Date and foreign databases such as PubMed and the Web of Science from their inception to November 2023. The retrieval method adopted the combination of medical subject headings terms and free terms. The keywords included “Urticaria,” “Chronic Urticaria,” “Rubella block,” “Addiction rash,” “Xiaofeng Powder”, and “Random.” All literature was reviewed by 2 investigators (Chenhao Bi and Yuqi Jia) independently. Any disagreement was resolved by consultation with the third researcher (Fengqin Wei).

### 2.2. Inclusion and exclusion criteria

#### 2.2.1. Inclusion criteria.

Inclusion criteria were as follows:

1. Study type: Randomized controlled trials of Xiaofeng powder in the treatment of CU in Chinese and English were included.
2. Research object: The subjects were clinically diagnosed CU patients. The age, sex, race, and region of the included patients were not limited.
3. Interventions: Experimental group: modified Xiaofeng powder. Control group: the use of antihistamine drugs (dosage, dosage form, administration times, and course of treatment were not limited).
4. Outcome indicators: Main outcome indicators: Cure rate, evaluation criteria including the Urticaria Activity Score specified in the Guidelines for Diagnosis and Treatment of Urticaria in China,^[4]^ and Symptoms and Signs Decline Index ^[5]^; Total effective rate = (total cases – invalid cases)/total cases × 100%; The recurrence rate was evaluated by the second clinical symptoms and signs during the follow-up period. Secondary outcome indicators: Adverse reaction rate; Symptoms and signs integral (itching degree; wind mass size; wind mass number; wind mass duration). Other outcome indicators: itch elimination time; disappearance time of rubella block; regression time of skin lesions; heat fade time; windbreaks; symptom relief rate; leukotriene level; main symptoms and signs; and serum total IgE, LTE4, LTB4, and HA levels.

#### 2.2.2. Exclusion criteria.

Exclusion criteria were as follows:

1. Non-randomized controlled trials
2. Repeated published literature
3. Unable to extract full data
4. No clear diagnostic criteria
5. No clear evaluation standard of curative effect
6. Dosage or dosage form not specified

### 2.3. Study selection and data extraction

The literature screening work was carried out by 2 researchers who extracted data, conducted cross-checking and reviewed the data according to the predetermined table. When there was a disagreement, it was determined by the third researcher. The data extraction content includes basic information about the included literature, outcome indicators, and quality evaluation information of the included indicators.

### 2.4. Risk of bias assessment

The bias risk was assessed according to Cochrane 5.1.0.

The assessment included the random allocation method, allocation concealment, the blinding method in the trial process and outcome data integrity. High-risk, low-risk, and uncertainty-risk assessments of included studies were based on the evaluation manual.

### 2.5. Statistical analysis and data synthesis for meta-analysis

Rev Man 5.3 was used for statistical analysis of literature data. The OR and its 95% CI were used to represent the effect amount in the enumeration data. The measurement data used the mean difference (MD) and its 95% CI to represent the effect. Heterogeneity among the included studies was assessed performed using the chi-squared test. If *P* ≥ .1 and I^2^ ≤ 50%, a fixed-effect model analysis was used. If there was statistical heterogeneity (*P* < .1, I^2^ > 50%), the source of heterogeneity was analyzed to determine whether a random effect model could be used. After sensitivity analysis or subgroup analysis according to its source, descriptive analysis was carried out if it was impossible to determine its source. For the main outcome indicators, if the included studies were ≥10, a funnel plot was used to qualitatively detect publication bias. Egger’s and Begg’s tests were used to quantitatively assess the potential publication bias.

## 3. Results

### 3.1. Description of studies

**Figure 1.**
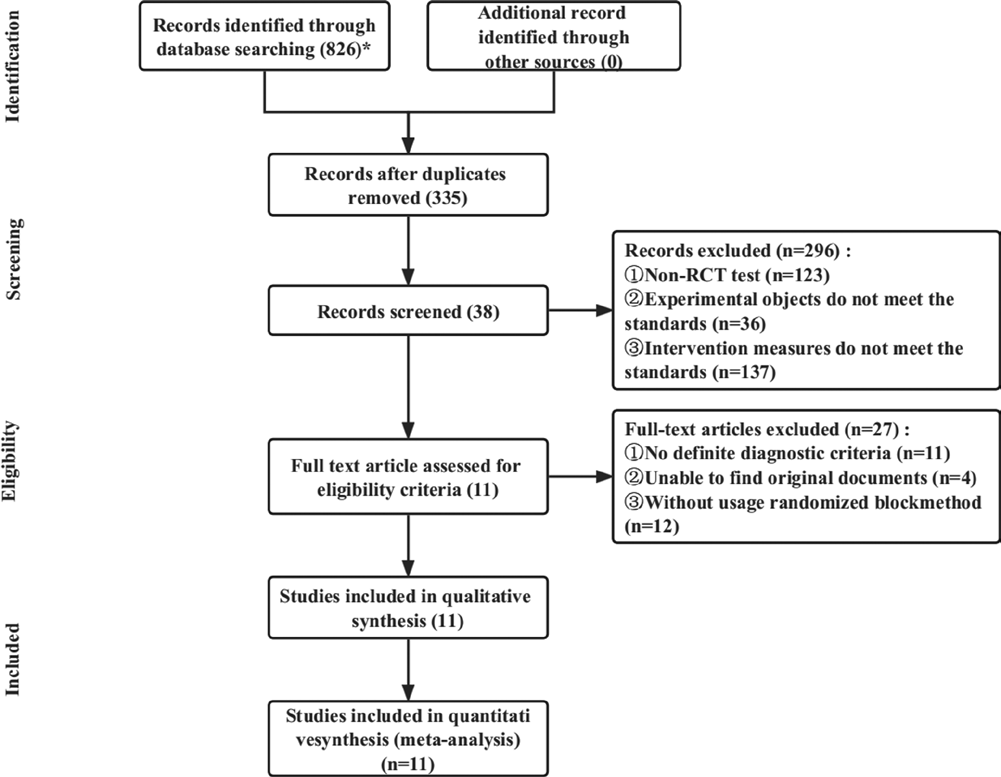
Flow chart of literature screening.* The number of retrieved studies and their databases are as follows: CNKI (n = 235), WanFang data (n = 232), CBM (n = 187), VIP (n = 170), PubMed (n = 2), and web of science (n = 0). CBM = China biomedical literature database, CNKI = China national knowledge infrastructure, VIP = China scientific journal database.

Of 826 related articles obtained by the initial search, 335 were obtained after the removal of duplicates. After reading the titles and abstracts, 38 articles were retained, and 27 were excluded after reviewing the full text. Finally, 11 studies were eligible for inclusion. The literature screening process is shown in Figure 1.

### 3.2. Characteristics of study

A total of 11 studies^[6–16]^ that enrolled 1076 patients included 550 patients in the experimental group and 526 patients in the control group. The treatment group was treated with Xiaofeng powder alone, while the control group was treated with antihistamine drugs. The basic characteristics of the included studies are given in Table 1.

**Table 1:** Basic characteristics of included studies.

Study	Sample size	Age	Sex	Intervention		Duration	Outcome
	(T/C)	(yr)	(M/F)	Trial group	control group	(d)	
Zhang X 2019^[6]^	40/40	17~75	49/31	Xiaofeng powder	Loratadine tablets	14	①②
Xuan XM 2019^[7]^	45/45	20~56	41/49	Xiaofeng powder	Cetirizine hydrochloride tablets	14	①②⑤
Yi JY 2018^[8]^	40/40	18~65	25/55	Xiaofeng powder	Loratadine tablets	42	①②③
Wu M 2018^[9]^	42/41	20~60	53/30	Xiaofeng powder	Levocetirizine hydrochloride oral liquid	28	③④
Chen KG 2017^[10]^	53/52	18~64	59/46	Xiaofeng powder	Levocetirizine hydrochloride oral liquid	28	①②③
Ma L 2016^[11]^	53/53	33.3	57/49	Xiaofeng powder	Cetirizine hydrochloride tablets	18~27	③④⑤
Guo J 2014^[12]^	80/80	18~64	68/52	Xiaofeng powder	Levocetirizine hydrochloride oral liquid	28	①②③
Ma JG 2013^[13]^	36/36	14~71	33/39	Xiaofeng powder	Mizolastine + chlorpheniramine	30	①②
Li M 2012^[14]^	49/49	20~48	51/47	Xiaofeng powder	Cetirizine hydrochloride tablets	10~30	③④⑤
Wu HB 2007^[15]^	70/50	19~70	44/76	Xiaofeng powder	Terfenadine + Cinnarizine	28	①②④
Xing JX 2003^[16]^	42/40	23~63	35/47	Xiaofeng powder	Chlorpheniramine + Cyproheptadine	42	①②

Note: outcome: ① Cure rate; ② Total effective rate; ③ Recurrence rate; ④ Adverse reaction rate; ⑤ Symptoms and signs integral (itching degree; wind mass size; wind mass number; wind mass duration).

### 3.3. Risk of bias assessment

**Figure 2.**
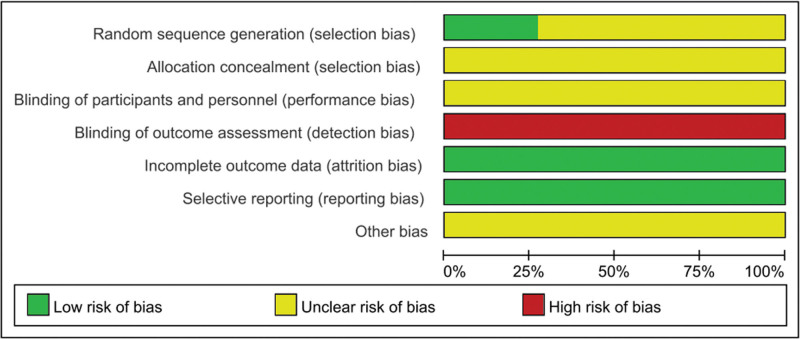
Risk of bias summary.

According to Cochrane 5.1.0, 11 included studies were assessed for bias risk. Regarding the random sequence generation method, 2 studies^[10,11]^ used the lottery grouping method, 1 study^[14]^ used the random number table method, and the other 8 studies only referred to random grouping. None of the studies^[6–16]^ mentioned the use of the blind method or other biases. All studies^[6–16]^ were assessed as having a high risk of bias in terms of the blinding of outcome assessment. All studies^[6–16]^ were assessed as having a low risk of bias in terms of incomplete outcome data and selective reporting. The results are shown in Figure 2.

### 3.4. Meta-analysis results

#### 3.4.1. Cure rate.

**Figure 3.**
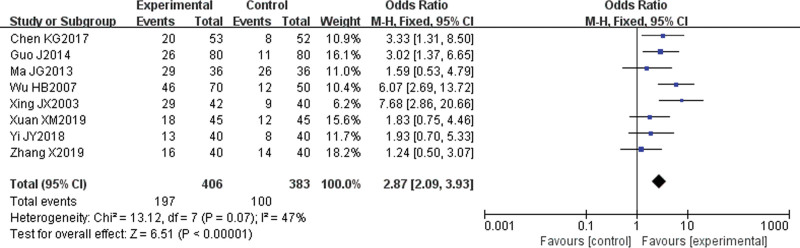
Meta-analysis forest map of the cure rate.

Eight papers^[6–8,10,12,13,15,16]^ reported cure rates. There was statistical heterogeneity among the studies (*P* = .07, I^2^ = 47%), and heterogeneity was not more than 50%; thus, the fixed effect model was used for meta-analysis. The results showed that the cure rate of the experimental group was higher than that of the control group, and the difference was statistically significant (odds ratio [OR] = 2.87, 95% CI [2.09, 3.93], *P* < .00001) (Fig. 3).

#### 3.4.2. Total efficiency rate.

**Figure 4.**
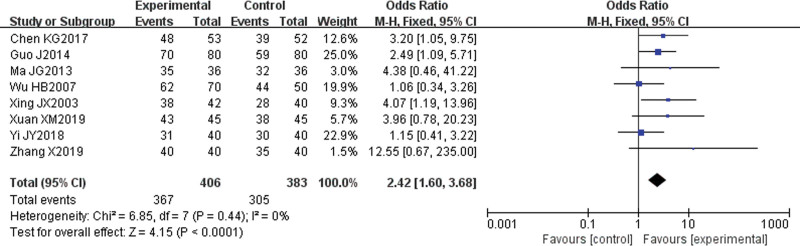
Meta-analysis forest map of the total effective rate.

Eight papers^[6–8,10,12,13,15,16]^ reported the total effective rates. There was no statistical heterogeneity among the studies (*P* = .44, I^2^ = 0%); therefore, a fixed-effect model was used for the analysis. The results showed that the total effective rate of the experimental group was higher (odds ratio [OR] = 2.42, 95% CI [1.60, 3.68], *P* < .0001), and the difference was statistically significant (Fig. 4).

#### 3.4.3. Recurrence rate.

**Figure 5.**
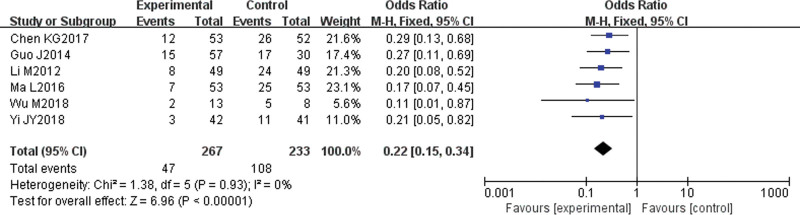
Meta-analysis forest map of recurrence rates.

Six studies^[8–12,14]^ reported recurrence rates. There was no statistical heterogeneity among the studies (*P* = .93, I^2^ = 0%); therefore, a fixed-effect model was used for the analysis. The results showed that the recurrence rate of patients in the experimental group was low (OR = 0.22, 95% CI [0.15, 0.34], *P* < .00001), and the difference was statistically significant (Fig. 5).

#### 3.4.4. Adverse reaction rate.

**Figure 6.**
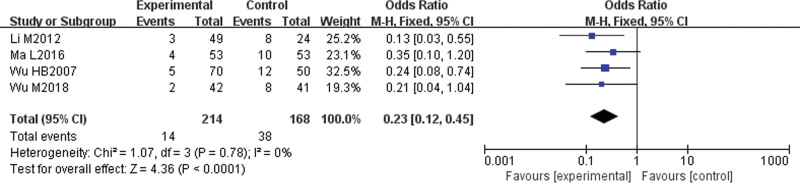
Meta-analysis forest map of adverse reaction rates.

Four studies^[9,11,14,15]^ reported adverse reaction rates. There was no statistical heterogeneity among the studies (*P* = .78, I^2^ = 0%); therefore, a fixed-effect model was used for the analysis. The results showed that the incidence of adverse reactions in the experimental group was lower (OR = 0.23, 95% CI [0.12, 0.45], *P* < .0001), and the difference was statistically significant (Fig. 6).

#### 3.4.5. Symptoms and signs integral (itching degree, wind mass size, wind mass number, wind mass duration).

**Figure 7.**
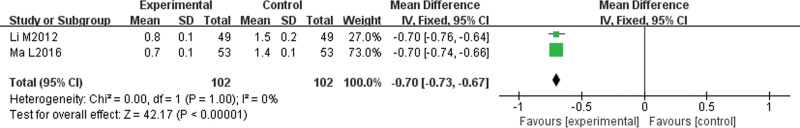
Meta-analysis forest map of itching degree.

**Figure 8.**
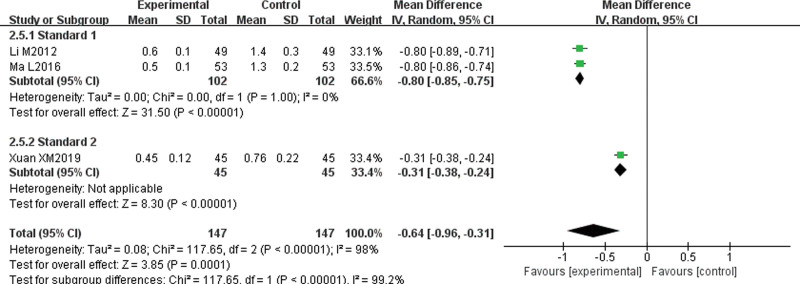
Meta-analysis forest map of wind mass size.

**Figure 9.**
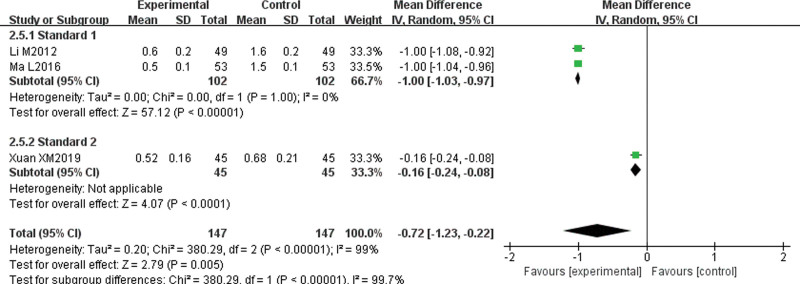
Meta-analysis forest map of wind mass number.

**Figure 10.**
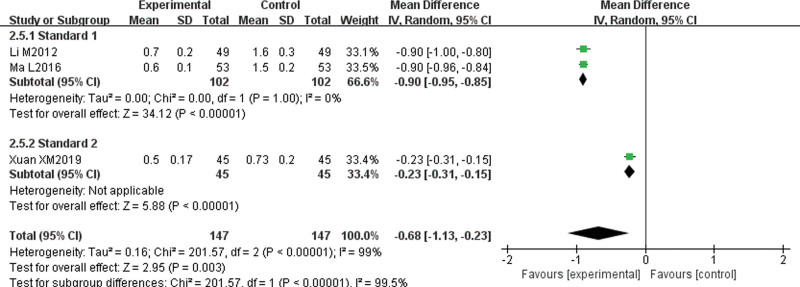
Meta-analysis forest map of wind mass duration.

Three papers^[7,11,14]^ reported symptom and sign integrals. These integrals were divided into “Standard 1” group and “Standard 2” group according to integral standards. There was no statistical heterogeneity among the studies about itching degree (*P* = 1.00, I^2^ = 0%); therefore, a fixed-effect model was used for the analysis. The results showed that the degree of itching in the experimental group was lower than that in the control group, and the difference was statistically significant (MD = −0.70, 95% CI [−0.73, −0.67], *P* < .00001). There was great statistical heterogeneity among the studies about the size of wind mass (*P* < .1, I^2^ = 98%), the number of wind mass (*P* < .1, I^2^ = 99%), and the duration of wind mass (*P* < .1, I^2^ = 99%), and heterogeneity were more than 50%; therefore, the random-effect model was used for subgroup analysis. The results showed that the wind mass size (MD = −0.64, 95% CI [−0.96, −0.31], *P* = .0001), wind mass number (MD = −0.72, 95% CI [−1.23, −0.22], *P* = .005), and wind mass duration (MD = −0.68, 95% CI [−1.13, −0.23], *P* = .003) in the experimental group were lower than those in the control group, and the difference was statistically significant. There was no significant decrease in heterogeneity after subgroup analysis, which suggests that different scoring criteria may not be the main cause of heterogeneity (Figs. 7–10).

### 3.5. Other outcome indicators

Due to differences in the evaluation indicators of study results and because the data provided by individual studies were not specific enough, a meta-analysis of all outcome indicators was not carried out, so the following outcome indicators were descriptively analyzed. Xuan XM^[7]^ reported that the regression time of pruritus, rubella, skin lesions, and burning in the experimental group was significantly shorter than those in the control group. Yi JY^[8]^ reported that patients in the experimental group had a lower incidence of wind mass attacks after treatment. Wu M^[9]^ reported that the symptom relief rate and leukotriene level improvement in the experimental group were better than those in the control group after treatment. Guo J^[12]^ reported that the improvements in the main symptoms and signs were better in the experimental group. Ma JG^[13]^ reported that the total levels of serum for IgE, LTE4, LTB4, and HA were lower in the experimental group than in the control group.

### 3.6. Sensitivity analysis

Sensitivity analysis of the above indicators was performed, removing the included studies one by one. The results showed no fundamental changes, indicating high reliability.

### 3.7. Publication bias

Egger’s test and Begg’s test were used to evaluate whether there was publication bias in the main outcome indicators. The results showed that no evidence of publication bias was found in the cure rate (Egger’s test *P* = .500 > .05, Begg’s test *P* = .711 > .05), the total effective rate (Egger’s test *P* = .168 > .05, Begg’s test *P* = .174 > .05) or the recurrence rate (Egger’s test *P* = .146 > .05, Begg’s test *P* = .060 > .05).

## 4. Discussion

At present, the pathological mechanism of CU is not clear, and it is considered that the allergic reaction of the body under the action of special factors leads to local capillary congestion and expansion and inflammatory reaction. Western medicine in the treatment of more use of antihistamines combined with immunosuppressive agents or the use of a variety of desensitization drug combination therapy.^[17]^ However, urticaria has not been cured completely, and there are some problems such as limited effect, high price of some drugs, and various adverse reactions.^[18]^

TCM considers that CU is caused by external attack of wind-cold pathogens, the disharmony of Ying and Wei, or extreme internal heat wind and blood deficiency wind. Therefore, TCM treatment is mostly based on wind pathogens. Xiaofeng powder has been shown to have anti-inflammatory, antipruritic, immune regulatory, and antiallergic effects^[19]^ and can reduce the total serum IgE level^[13]^ and serum leukotriene level^[9]^ in patients with CU. Modern pharmacology found that Schizonepeta tenuifolia has good antiallergic effects and can effectively inhibit acute and chronic inflammation.^[20]^ The antiallergic effect of windbreak can be achieved by inhibiting PAR-2 expression and then inhibiting mast cells.^[21]^ Cicada molting has the function of inhibiting nonspecific immunity and has good heat-clearing and sterilization effects^[22]^; Sophora flavescens is a commonly used drug in the treatment of skin diseases in TCM and has anti-inflammatory and sterilization effects.^[23]^

In this paper, 11 randomized controlled trials were included in the meta-analysis to provide reliable evidence for the clinical use of Xiaofeng powder in the treatment of CU. In terms of improving the cure rate and treatment efficiency, the efficacy of Xiaofeng powder in the experimental group was significantly higher than that of antihistamine drugs in the control group. In the integral of symptoms and signs, although the integral standards were different, the clinical effect of Xiaofeng powder treatment was better than that of antihistamines. The experimental group had a better effect than the control group in reducing the recurrence and adverse reaction rates. All the above results have statistical and clinical significance, and the data results are reliable. Therefore, Xiaofeng powder has better clinical efficacy and higher safety than antihistamine drugs in the treatment of CU.

This study has several limitations: The methodological quality of the included studies was low. The number of included studies was small, no reference was made to the implementation of allocation concealment and blinding methods, and there were problems such as risk offset. All subjects included in this study lived in China, and there may be regional restrictions.

## 5. Conclusion

This meta-analysis demonstrated that, compared with antihistamine drugs, Xiaofeng powder has a better curative effect and higher safety in the treatment of CU. However, due to the limitations of this study, it is necessary to pay attention to the reports of adverse reactions and negative results in future clinical studies, and more high-quality studies with large sample sizes are needed to further verify the conclusions of this study.

## Author contributions

**Conceptualization:** Chen Hao Bi, Fengqin Wei.

**Data curation:** Chen Hao Bi, Yuqi Jia.

**Formal analysis:** Chen Hao Bi, Yuqi Jia.

**Funding acquisition:** Fengqin Wei.

**Investigation:** Chen Hao Bi, Yuqi Jia.

**Methodology:** Chen Hao Bi, Yuqi Jia.

**Project administration:** Fengqin Wei.

**Resources:** Fengqin Wei.

**Software:** Chen Hao Bi, Yuqi Jia.

**Supervision:** Fengqin Wei.

**Validation:** Yuqi Jia, Fengqin Wei.

**Visualization:** Chen Hao Bi, Yuqi Jia.

**Writing – original draft:** Chen Hao Bi.

**Writing – review & editing:** Yuqi Jia, Fengqin Wei.
